# The influence of chronic exercise intervention on the executive function of children with neurodevelopmental disorders: a meta-analysis

**DOI:** 10.3389/fneur.2026.1827768

**Published:** 2026-06-29

**Authors:** Zhengyang Zhao, Zhihao Feng, Jiaxin Deng, Yongfeng Liu

**Affiliations:** School of Sports Training, Chengdu Sport University, Chengdu, Sichuan, China

**Keywords:** children, chronic exercise, executive function, meta-analysis, neurodevelopmental disorders

## Abstract

**Background:**

This meta-analysis examined whether chronic exercise interventions can improve executive function in children with neurodevelopmental disorders and aimed to identify the conditions under which these benefits are most pronounced. Although the review was framed within the broader category of neurodevelopmental disorders, the available randomized controlled evidence was mainly concentrated on attention-deficit/hyperactivity disorder (ADHD) and autism spectrum disorder (ASD). Therefore, the findings should be interpreted primarily in relation to these two diagnostic groups rather than the full spectrum of neurodevelopmental disorders.

**Methods:**

A systematic search of six major databases was conducted to identify randomized controlled trials examining the effects of chronic exercise interventions on executive function in children with neurodevelopmental disorders. Thirteen randomized controlled trials involving 527 children were included, comprising 10 studies on children with ADHD and 3 studies on children with ASD. Executive function outcomes were synthesized across three core domains: inhibitory control, working memory, and cognitive flexibility. Effect sizes were pooled using a random-effects model, and subgroup analyses were performed according to exercise type, intervention duration, exercise intensity, diagnostic subtype, and control condition.

**Results:**

The pooled results showed that chronic exercise produced a moderate overall improvement in executive function. Among the three executive function domains, the largest effect was observed for inhibitory control, followed by a smaller but significant improvement in working memory. Cognitive flexibility also showed a small but statistically significant benefit after harmonization of outcome direction. Subgroup analyses indicated that intervention effects varied across exercise type, duration, intensity, diagnostic subtype, and control condition. In particular, closed-skill exercise appeared to produce more stable benefits for inhibitory control and working memory. Longer interventions were more favorable for improving inhibitory control, while moderate-to-higher intensity exercise showed greater promise for working memory.

**Discussion:**

These findings suggest that chronic exercise may be a promising and accessible non-pharmacological approach for supporting executive function in children with ADHD and ASD. The results further indicate that exercise should not be regarded as a uniform intervention for executive function, because its effects differ across cognitive subdomains and intervention characteristics. Conceptually, this study advances the field by moving beyond the general question of whether exercise is effective and instead clarifies how, for whom, and for which aspects of executive function exercise may be most beneficial. However, the findings should be interpreted with caution because of substantial between-study heterogeneity and the limited number of trials included in several subgroup analyses.

**Systematic review registration:**

https://www.crd.york.ac.uk/PROSPERO/search, identifier CRD420261329177.

## 1 Introduction

Neurodevelopmental disorders (NDDs) refer to a range of conditions that arise during early brain development, primarily including attention-deficit/hyperactivity disorder (ADHD), autism spectrum disorder (ASD), learning disabilities, developmental language disorders, and intellectual developmental disorders (1). These disorders usually begin in childhood and can have a lasting impact on an individual’s cognition, emotions, behaviors, and social functioning, constituting a significant global public health burden. According to the Global Burden of Disease 2021 study, the prevalence of ASD among children aged 0–14 increased from 1990 to 2021, while the prevalence of ADHD slightly decreased by 0.08% (2). However, there are significant differences in the distribution of disease burden among different countries and regions. The burden of ASD and ADHD is more prominent in areas with a high sociodemographic index, while the burden of intellectual development disorders is more prominent in areas with a low sociodemographic index (2). Regional studies have also confirmed this trend. A community-based epidemiological survey in India showed that the prevalence of NDDs among children under 12 years old was as high as 1.38%, among which ADHD, specific learning disabilities, and ASD were the main diagnostic subtypes. These data indicate that NDDs have become a significant health issue affecting children’s development, and effective early identification and intervention strategies are urgently needed (1). In this study, most included studies involved children with ADHD or ASD; therefore, the findings should primarily be generalized to these two diagnostic groups rather than to all neurodevelopmental disorders.

Executive function is a higher-order cognitive process that enables goal-directed behavior. Its key components are inhibitory control, working memory, and cognitive flexibility. Executive dysfunction commonly co-occurs in children with neurodevelopmental disorders and can profoundly affect their academic performance, social adaptation, and long-term quality of life (3). Across the world, ADHD is reported to affect roughly 5–7% of the population, whereas ASD is seen in about 1–2% of children, together representing a substantial public health burden (4). Notably, early difficulties in executive function and self-regulation strongly predict later attention, social communication, and adaptive functioning, highlighting the importance of executive function development for both early cognition and subsequent outcomes in children with NDDs (3). Some studies have conducted meta-analysis highlighting a significant longitudinal association between early executive and self-regulation difficulties and later impairments in attention, socio-communication, and adaptive functioning in NDDs (3). Furthermore, the developmental trajectories of executive function subdomains differ among individuals and are shaped by factors such as age, sex, diagnostic category, and the assessment instruments employed (5).

Currently, treatments for ADHD primarily include medication and exercise-based behavioral interventions. A meta-analysis of placebo-controlled trials found that all included medications were superior to placebo in alleviating core ADHD symptoms in children and adolescents, as evaluated by clinicians over approximately 12 weeks (6). Nevertheless, drug therapy is not suitable for every patient. Recent experimental studies indicate that about 20–30% of children with ADHD show little or no response to medication, and its use may be accompanied by side effects, limiting its clinical applicability (7). Given these constraints, behavioral and non-pharmacological interventions play a crucial role in the comprehensive management of NDDs. International guidelines suggest that for preschool-aged children with ADHD, parent-focused behavioral training and classroom interventions should be prioritized. For school-aged children over six, a combination of medication and behavioral or educational interventions is recommended (8).

In this context, chronic exercise interventions have attracted considerable attention for their beneficial effects on brain plasticity. Evidence suggests that exercise can enhance neural adaptability through various mechanisms, including increased release of brain-derived neurotrophic factors, improved cerebral blood flow, and modulation of neurotransmitter levels, all of which contribute to better executive function (9). Compared with pharmacological treatments, exercise offers advantages such as minimal side effects, greater accessibility, and easier implementation, making it especially valuable for children who cannot tolerate medications or respond poorly to them (10). Exercise also tends to be more engaging than purely behavioral interventions. Studies indicate that children are generally more receptive to programs that incorporate physical activity, which can boost adherence and long-term participation (11). Recent high-quality network meta-analyses have provided further evidence that exercise is effective in improving executive function in children with neurodevelopmental disorders. Notably, exercise play (SMD = 0.94) and multicomponent physical activity (SMD = 0.79) were associated with moderate to large effect sizes. Ball games have also been proven to be one of the most effective forms of exercise for improving executive function (12). However, the existing research results still show considerable heterogeneity, and systematic integration and in-depth analysis are urgently needed. Previous meta-analyses have demonstrated that physical activity has potential benefits for the executive function of patients with neurodevelopmental disorders, while also emphasizing the need to clarify the regulatory role of intervention methods and dosages (13). Although ADHD and ASD differ in their core clinical manifestations, both are classified as neurodevelopmental disorders and are frequently characterized by impairments in executive function, including inhibitory control, working memory, and cognitive flexibility. Therefore, combining these two diagnostic groups within a broader neurodevelopmental-disorder framework is theoretically justifiable when the research focus is executive function. Nevertheless, given the clinical heterogeneity between ADHD and ASD, diagnostic subtype was examined as a subgroup variable, and the results were interpreted cautiously rather than generalized to all neurodevelopmental disorders. Therefore, the present meta-analysis systematically evaluated the impact of chronic exercise intervention on the executive function of children with neurodevelopmental disorders through a meta-analysis, down to three subfields: inhibitory control, working memory, and cognitive flexibility were evaluated. Subgroup analyses were conducted from multiple perspectives, such as intervention type, intervention duration, exercise intensity, diagnostic subtype, and control group type, to comprehensively explore the potential regulatory factors influencing the intervention effect.

## 2 Materials and methods

This meta-analysis was conducted in accordance with the Preferred Reporting Items for Systematic Reviews and Meta-Analyses (PRISMA) 2020 statement (14) and the Cochrane Handbook for Systematic Review of Interventions (version 6.5) (15). The protocol was prospectively registered in PROSPERO (CRD420261329177). It also provides an overview of the effects and extent of chronic exercise interventions on executive function in children with neurodevelopmental disorders, with the aim of offering evidence to support strategies for improving executive function in this population.

### 2.1 Search strategy

A systematic literature search was conducted from database inception to January 24, 2026, in PubMed, Web of Science, EBSCO, the Cochrane Library, Embase, and PsycINFO. The core search terms included physical exercise, chronic exercise, executive function, children with neurodevelopmental disorders, and randomized controlled trials. Controlled vocabulary and free-text terms were combined using Boolean operators. The full search strategy for each database is provided in Supplementary Table S1. In addition, Google Scholar and the reference lists of all included studies and relevant reviews were manually screened to identify potentially eligible studies.

### 2.2 Qualification criteria and selection

We included studies that met all of the following criteria: (1) Participants: We included children under the age of 15 with neurodevelopmental disorders. Children were eligible if they had been diagnosed with a neurodevelopmental disorder according to established diagnostic criteria, such as DSM-IV, DSM-5, ADOS/ADOS-2, or ICD-10. (2) Intervention measures: The experimental group received various forms of exercise intervention, such as aerobic exercise, non-aerobic exercise (motor skills training, coordination training, and cognitive training), and mixed exercise. In the present review, the term “chronic exercise intervention” was used operationally to distinguish repeated, structured exercise programs from acute single-session exercise. Therefore, studies were eligible if the exercise intervention lasted at least 8 weeks. We acknowledge that this definition is broader than a stricter long-term threshold, such as 6 months. To avoid overinterpretation, intervention duration was further examined through subgroup analysis, and the implications of short-duration interventions were interpreted cautiously in the Discussion and Limitations sections. (3) Outcome measures: Executive function (inhibitory control, working memory, and cognitive flexibility). (4) Research Design: Randomized controlled trial design. The following studies were excluded: (1) the non-English literature, dissertations, conference summaries, and review articles. (2) Republished research. (3) Missing data, incorrect or missing information, or inability to extract. (4) Research involving adults and animals.

### 2.3 Data extraction

Two reviewers (ZYZ and ZHF) independently completed the study selection and data extraction process using EndNote 20. When discrepancies arose, they were resolved through discussion with a third reviewer (YFL). During screening, ZHF first assessed the titles and abstracts, and ZYZ then examined the full texts for eligibility. The final data analysis was carried out by ZYZ, ZHF, and JXD, with oversight and final review provided by YFL. For studies in which the required data could not be obtained directly, we attempted to contact the corresponding authors. If the necessary information remained unavailable, those studies were not included in the quantitative synthesis. The data were summarized into a table as shown in Table 1. The data in the table include the author (year), country or region, participant characteristics (sample characteristics, age, symptom diagnosis method, sample size), intervention measures (intervention item, intervention type, intervention frequency, intervention intensity), outcome indicators (domain, measurement tool), and the control group.

**Table 1:** Study characteristics.

Author (year)	Country	Participant characteristics			Intervention measure				Outcome (type: tools)	control group
		Sample characteristics; age (mean)	Diagnostic methods	Sample size (IG/CG)	Program	Type	Frequency (time-duration)	Intensity		
Ahn et al. (30)	South Korea	ADHD;9	DSM-5, Korea version K-SADS-PL	23/23	Equine-assisted activities	Open	40 min- 4 months, 2 per week	M	IC: CPT	NT
Alooche et al. (28)	Iran	ASD;9	GARS-3	15/15	Physical training	Close	45 min- 2 months, 3 per week	MTV	IC/WM: BRIEF CF: WCST	TAU
Benzing and Schmidt (26)	Switzerland	ADHD;10.63	ICD-10	28/23	Exergaming	Open	30 min - 2 months, 3 per week	MTV	IC: Simon task WM: CSB CF: Flanker	NT
Chang et al. (20)	China	ADHD;8.36	DSM-5	IG1:16/IG2:16/16	Table tennis	Close	60 min- 3 months, 3 per week	M	IC: SCWT CF: WCST	NT
Chen et al. (21)	China	ASD;5.17	DSM-5, ADOS-2	15/15	Sports games	Open	30 min - 2 months, 6 per week	M	IC: Day/Night Task WM: DSK CF: DCCS	TAU
Deng et al. (22)	China	ASD;7.84	DSM-5	12/12	Gymnastics exercises	Close	40 min - 3 months, 3 per week	M	IC: DNST WM: SOPT CF: WCST	TAU
Huang et al. (23)	China	ADHD; 10.21	DSM-4, Chinese ADHDT, CBCL	12/12	Inline skating	Open	80 min-3 months, 2 per week	M	IC: SCWT WM: CANTAB	NT
Kadri et al. (31)	Tunisia	ADHD; 14.5	NR	20/20	Taekwondo	Open	50 min-18 months, 2 per week	NR	IC: SCWT	TAU
Liang et al. (24)	China	ADHD; 8.46	DSM-5	40/40	Skipping, cardio kickboxing, agility ladder, basketball, table tennis, and badminton	Open	60 min-3 months, 3 per week	MTV	IC: Flanker WM: TOL CF: TMT	NT
Ludyga et al. (27)	Switzerland	ADHD; 10.4	DSM-5	29/28	Judo	Open	60 min-3 months, 2 per week	M	WM: K-score	NT
Memarmoghaddam et al. (29)	Iran	ADHD; 8.3	SNAP-IV, CBCL	19/17	Table tennis, target games, bowling, balance beam, station training, treadmill running, ball games.	Close	90 min-2 months, 3 per week	MTV	IC: GNG	NT
Pan et al. (25)	China	ADHD; 9	DSM-4, Chinese CBCL and ADHDT	16/16	Table tennis	Open	70 min-3 months, 2 per week	MTV	IC: SCWT	NT
Ziereis and Jansen (17)	Germany	ADHD;9.45	ICD-10	13/16	IG1:skill-based; IG2:general sports and games	Open	60 min-3 months, 1per week	NR	WM:DST	NT

ASD, autism spectrum disorder; ADHD, attention deficit hyperactivity disorder; min, minutes; IG, intervention group; CG, control group; M, moderate; MTV, moderate-to-vigorous; AE, aerobic exercise; NAE, not aerobic exercise; NR, no report; DSM-4, Diagnostic and Statistical Manual of Mental Disorders, Fourth Edition; IC, inhibitory control; WM, working memory; CF, cognitive flexibility; SCWT, Stroop Color and Word Test; CSB, color span backwards; CANTAB, Cambridge Neuropsychological Test Automated Battery; AC, active control; BDST, Backward Digit Span Test; HRmax, percentage of maximal heart rate; DCCS, Dimensional Change Card Sorting; ADHD RS-IV, ADHD Rating Scale-IV; KiTAP, Kinder Test of Attentional Performance; DNST, Day-Night Stroop Task; SOPT, Self-Ordered Pointing; WCST, Wisconsin Card Sorting Test; CBCL, Child Behavior Checklist; HRR, Heart Rate Reserve; GNG, Go-No-Go; NI, no intervention; CBCL, Child Behavior Checklist; IEP, Individualized Education Plan; ADOS, Autism Diagnostic Observation Schedule; Hayling, The Junior Hayling Test; ADOS-2, Autism Diagnostic Observation Schedule,2nd Edition; ADI-R, Autism Diagnostic Interview-Revised; SCQ Social Communication Questionnaire; TMT, Trail Making Test; TOL, Tower of London test; BRIEF-II, Behavior Rating Inventory of Executive Function, 2nd Edition; C-WISC, Wechsler Intelligence Scale for Children Chinese revised; FMS, Fundamental Movement Skills; DST, Digit span task; ICD-10, International Classification of Diseases, Tenth Revision; K-SADS-PL, Kiddie-Schedule for Affective Disorders and Schizophrenia Present and Lifetime; CPT, Conners’ Continuous Performance Test 2nd Edition; NT, no treatment; TAU, treat as usual. Ahn et al. (30), Alooche et al. (28), Benzing and Schmidt (26), Chang et al. (20), Chen et al. (21), Deng et al. (22), Huang et al. (23), Kadri et al. (31), Liang et al. (24), Ludyga et al. (27), Memarmoghaddam et al. (29), Pan et al. (25), and Ziereis and Jansen (17).

Since the 13 included studies did not provide a comprehensive score for the overall concept of executive function, we classified and extracted the outcome measures based on the core components of executive function (inhibitory control, working memory, and cognitive flexibility). The classification is based on the unified model of executive function proposed by Miyake et al. (16) and combined with the actual test contents of the measurement tools used in each research institute. In the overall executive function analysis, some studies contributed more than one effect size when multiple executive-function domains were reported. Therefore, the pooled estimate for overall executive function was intended to provide a broad summary across executive-function domains rather than a strictly independent study-level estimate. Separate meta-analyses were also conducted for each core domain to provide more specific evidence for inhibitory control, working memory, and cognitive flexibility. For each study, we extracted the mean (mean), standard deviation (SD), and total sample value (total) of each group (experimental group/control group) after the intervention. For studies that did not directly report the mean and standard deviation in the original text and only presented the results in graphical form, this study used image digital extraction tools to extract the data, and two researchers independently completed the input and verification. In case of any disagreement, it shall be resolved through discussion and consultation. If necessary, the decision shall be made by a third researcher. To reduce estimation errors, this study further examined the impact of graphic extraction data on the overall results through sensitivity analysis (17). After completing the extraction of the above basic data, we used RevMan 5.3 software (18) to enter the mean, standard deviation, and sample size of the intervention group and the control group included in the study. As different studies employ a variety of neuropsychological testing tools to assess various domains of executive function and have different measurement units, we calculated the standardized mean difference (SMD) as an effect size indicator. To correct for the possible bias brought by small-sample studies, Hedges’ g was adopted as the estimated value of SMD. To ensure consistency across outcome measures, all executive function scores were harmonized so that effect sizes in the same direction reflected better post-intervention performance. For cognitive flexibility, negative SMD values indicate improvement after score transformation. The heterogeneity analysis indicated substantial variability across the included studies, suggesting that the observed differences in effect sizes were greater than would be expected from sampling error alone. Given the clinical and methodological heterogeneity of the included studies in terms of intervention protocols (type, duration, intensity), sample characteristics (age, diagnostic subtype), and measurement tools, we pre-selected a random-effects model for meta-analysis to accommodate this high heterogeneity.

### 2.4 Research quality assessment

Researchers ZYZ and ZHF, respectively, used the Cochrane bias risk assessment tool (19) to conduct individual quality assessments of the included studies. During this process, in-depth discussions should be held on the differences that emerged in the assessment to seek consensus. If there are still differences among the reviewers after the discussion, a third reviewer will be invited to participate in the adjudication to ensure the objectivity and accuracy of the research results. The methodological quality of all included studies was assessed using the Cochrane Risk of Bias Tool, which examines seven domains: random sequence generation (selection bias), allocation concealment (selection bias), blinding of participants and personnel (performance bias), blinding of outcome assessment (detection bias), incomplete outcome data (attrition bias), selective reporting (reporting bias), and other potential biases. Based on responses to the signaling questions, each domain was rated as “low risk of bias,” “unclear risk of bias,” or “high risk of bias,” facilitating an overall bias assessment for each study.

## 3 Results

### 3.1 Literature screening process and the result

**Figure 1:**
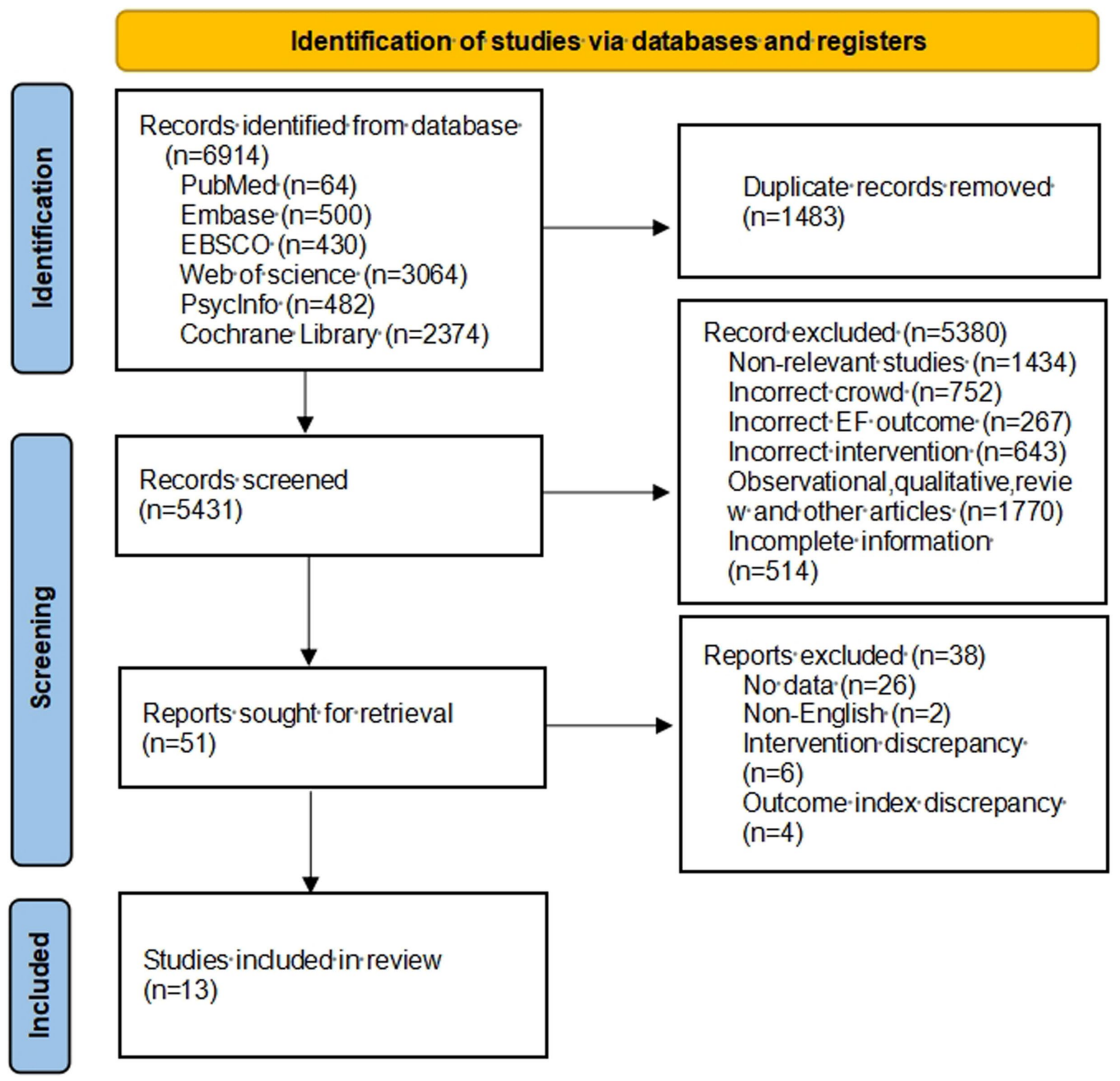
PRISMA flows study selection diagram.

The database search initially identified 6,914 records. Among them, 2,374 were retrieved from the Cochrane Library, 64 from PubMed, 3,064 from Web of Science, 500 from Embase, 430 from EBSCO, and 482 from PsycInfo. All records were then imported into EndNote 20 for reference management, and 1,483 duplicates were removed. This left 5,431 records for further review. Following title and abstract screening, 5,380 studies were excluded based on the predefined exclusion criteria. These excluded studies mainly consisted of irrelevant articles (*n* = 1,434), studies involving non-target populations (*n* = 752), and studies that did not assess executive function outcomes (*n* = 267); non-physical exercise intervention measures (*n* = 643); observational, qualitative, review, and other types of articles (*n* = 1770); and the lack of information (*n* = 514). The full texts of the remaining 51 studies were reviewed, and eliminate 38 studies that do not conform, including those without specific data (*n* = 26); non-English (*n* = 2); intervention differences, non-chronic exercise (*n* = 6); and the difference in the numerical values of the outcome indicators (*n* = 4). A total of 13 studies were finally screened out and included. The literature search results and research screening process are shown in Figure 1.

### 3.2 Study characteristics

Table 1 summarizes the main characteristics of the studies included in this review. A total of 13 randomized controlled trials were identified, and each study reported outcome data for at least one of the three core components of executive function: inhibitory control (IC), working memory (WM), and cognitive flexibility (CF). Because the included evidence was dominated by ADHD studies, the overall pooled results should be interpreted as being driven primarily by ADHD samples. The ASD-related findings were retained because they met the predefined eligibility criteria and shared the target outcome of executive function, but they should be considered exploratory due to the limited number of ASD trials. Among them, 6 studies were conducted in China (20–25), 2 in Switzerland (26, 27), 2 in Iran (28, 29), 1 in South Korea (30), 1 in Tunisia (31), and 1 in Germany (17). The total number of participants in the study was 527, with 274 in the experimental group and 253 in the control group. The diagnostic population of 10 studies (17, 20, 23–27, 29–31) was ADHD, and that of 3 studies (21, 22, 28) was ASD. All the participants included were children. The shortest exercise intervention period was 8 weeks, and the longest was 72 weeks. Two studies (31) did not report the intensity of exercise intervention, six studies (20–23, 27, 30) reported a moderate intensity of exercise intervention, and five studies (24–26, 28, 29) reported a moderate to high intensity of exercise. The various types of sports involved in the intervention include physical training, gymnastics, table tennis, equestrianism, skills training, skating, judo, badminton, and sports games. Nine studies used open-skill exercise interventions (17, 21, 23–27, 30, 31) and four studies on closed-skill exercise (20, 22, 28, 29). Compared with the experimental group, the control group included prolonged sitting without intervention (17, 20, 23–27, 29, 30) and maintaining regular training (21, 22, 28, 31).

### 3.3 Risk of bias

**Figure 2:**
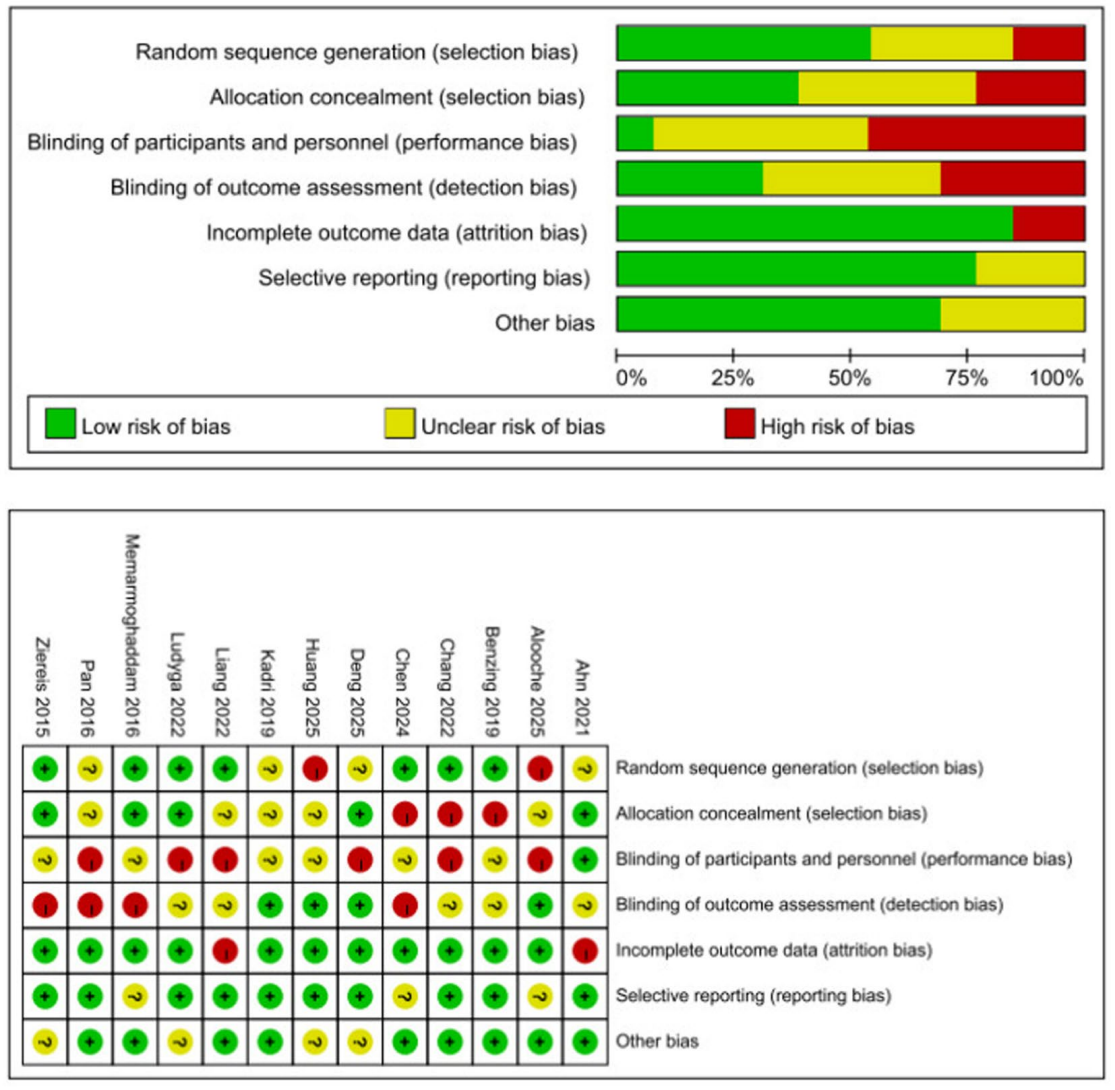
Results of the Cochrane risk of bias tool.

The methodological quality of the included studies was evaluated using the Cochrane Risk of Bias Tool, which covers seven domains. With regard to random sequence generation, 7 studies (17, 20, 21, 24, 26, 27, 29) were judged as low risk, 4 (22, 25, 30, 31) as unclear risk, and 2 (23, 28) as high risk. In terms of allocation concealment, five studies (17, 22, 27, 29, 30) were classified as low-risk, five studies (23–25, 28, 31) as unclear risk, and three studies (20, 21, 26) as high-risk. Regarding performance bias, one (30) study was identified as low-risk, six studies (17, 21, 23, 26, 29, 31) as unclear risk, and six studies (20, 22, 24, 25, 27, 28) as high-risk. Regarding detection bias, 4 studies (22, 23, 28, 31) were rated as low risk, 5 (20, 24, 26, 27, 30) as unclear risk and 4 (17, 21, 25, 29) as high risk. For loss to follow-up bias, 11 studies (17, 20–23, 25–29, 31) were assessed as low risk, and 2 (24, 30) as high risk. As for reporting bias, 10 studies (17, 20, 22–27, 30, 31) were classified as low risk, and 3 (21, 28, 29) as unclear risk. Finally, for other potential biases, 9 studies (20, 21, 24–26, 28–31) were considered low-risk, and 4 (17, 22, 23, 27) were of unclear risk, as shown in Figure 2.

### 3.4 Meta-analysis results

#### 3.4.1 Meta-analysis summary

**Figure 3:**
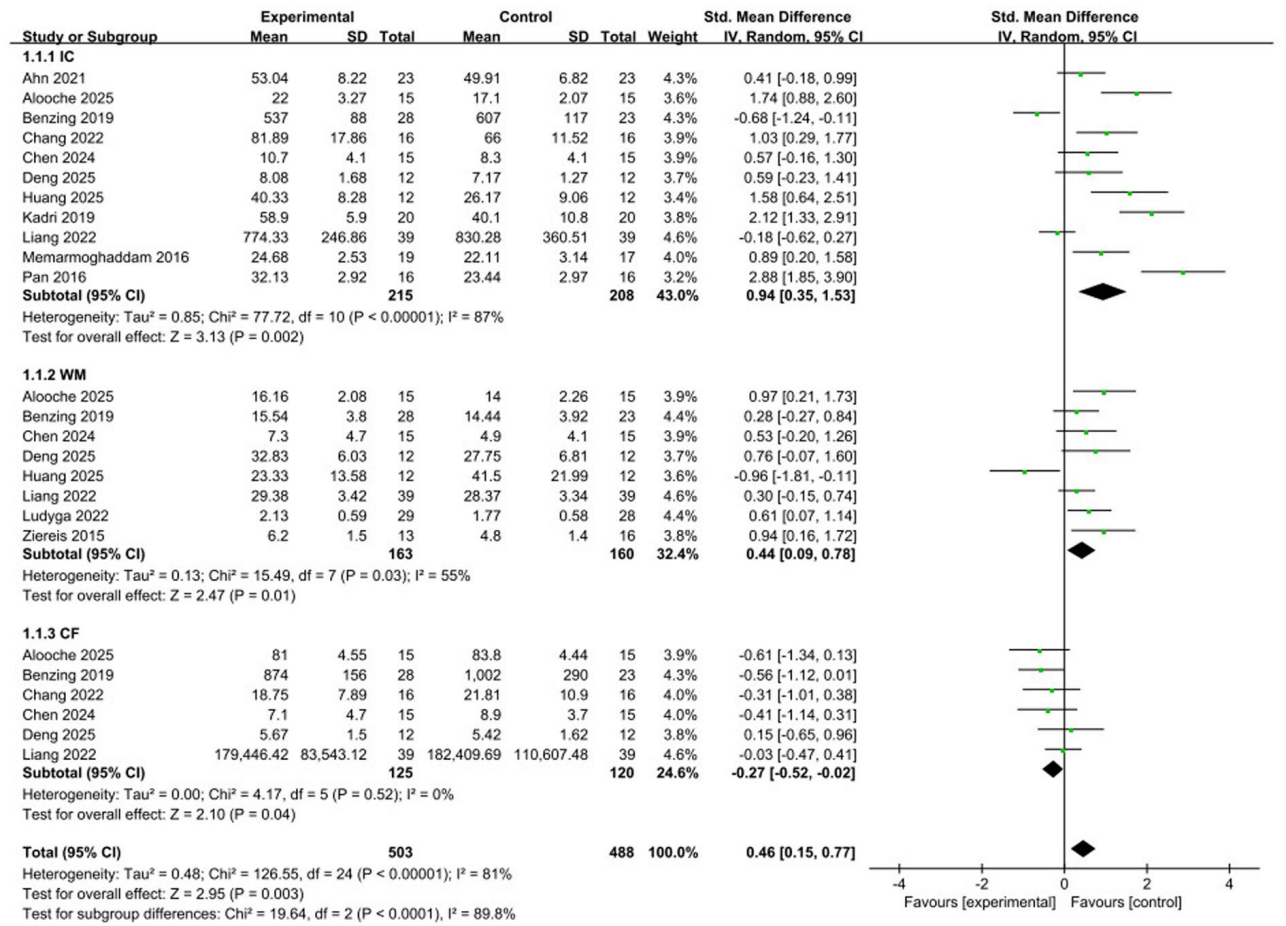
Forest plot of the effects of exercise intervention on EFs.

Since the 13 included studies did not provide a comprehensive score for the overall concept of executive function, we classified the data of the outcome measures based on the core components of executive function (inhibitory control, working memory, and cognitive flexibility). As shown in Table 2, there are a total of 25 effect sizes. Eleven studies provided data on inhibitory control (20–26, 28–31), eight studies provided data on working memory (17, 21–24, 26–28), and six studies provided data on cognitive flexibility (20–22, 24, 26, 28). Because some studies reported more than one executive-function outcome, the pooled estimate for overall executive function should be interpreted as a general summary across domains. As shown in Figure 3, the random-effects meta-analysis indicated that chronic exercise was associated with a moderate improvement in executive function among children with neurodevelopmental disorders (SMD = 0.46, 95% Cl: 0.15–0.77, *p* = 0.003), but there was a high degree of heterogeneity among the studies (*I*^2^ = 81%). This indicates considerable between-study variability, and the pooled estimate should therefore be interpreted with caution. Considering the differences among the subdomains of executive function, we further conducted subgroup analyses based on the three core domains and potential moderating variables. To improve the readability of the main text, detailed subgroup forest plots and funnel plots are provided as Supplementary Figures S1–5.

**Table 2:** Subgroup analysis of the effects of exercise intervention on executive functions.

Moderator	Domains	Subgroup analysis	*K*	SMD (95%Cl)	*p-*value	*I* ^2^	Total			P for interaction
							SMD (95%Cl)	*P*-value	*I* ^2^	
All studies	**–**	**–**	13	–	–	–	0.46 (0.15, 0.77)	*p* = 0.003	81%	–
Exercise type (2 sub-group analysis)	IC	Open	7	0.90 (0.05, 1.75)	*p* = 0.04	91%	0.94 (0.35, 1.53)	*p* = 0.002	87%	*p* = 0.77
		Close	4	1.04 (0.60, 1.48)	*p*<0.01	23%				
	WM	Open	6	0.32 (−0.08, 0.72)	*p* = 0.12	60%	0.44 (0.09, 0.78)	*p* = 0.01	55%	*p* = 0.12
		Close	2	0.87 (0.31, 1.44)	*p* = 0.002	0%				
	CF	Open	3	−0.27 (−0.61, 0.07)	*p* = 0.11	13%	−0.27 (−0.52, −0.02)	*p* = 0.04	0%	*p* = 0.99
		Close	3	−0.28 (−0.71, 0.15)	*p* = 0.20	0%				
Diagnostic subtype (2 sub-group analysis)	IC	ADHD	8	0.95 (0.19, 1.71)	*p* = 0.01	90%	0.94 (0.35, 1.53)	*p* = 0.002	87%	*p* = 1
		ASD	3	0.95 (0.21, 1.68)	*p* = 0.01	60%				
	WM	ADHD	5	0.28 (−0.2, 0.75)	*p* = 0.25	67%	0.44 (0.09, 0.78)	*p* = 0.01	55%	*p* = 0.16
		ASD	3	0.75 (0.3, 1.19)	*p* = 0.001	0%				
	CF	ADHD	3	−0.25 (−0.57, 0.07)	*p* = 0.13	7%	−0.27 (−0.52, −0.02)	*p* = 0.04	0%	*p* = 0.82
		ASD	3	−0.31 (−0.75, 0.12)	*p* = 0.16	0%				
Control group (2 sub-group analysis)	IC	NT	7	0.77 (0.03, 1.52)	*p* = 0.04	89%	0.94 (0.35, 1.53)	*p* = 0.002	87%	*p* = 0.39
		TAU	4	1.25(0.46, 2.03)	*p* = 0.002	74%				
	WM	NT	5	0.28 (−0.20, 0.75)	*p* = 0.25	67%	0.44 (0.09, 0.78)	*p* = 0.01	55%	*p* = 0.16
		TAU	3	0.75 (0.3, 1.19)	*p* = 0.001	0%				
	CF	NT	3	−0.25 (−0.57, 0.07)	*p* = 0.13	7%	−0.27 (−0.52, −0.02)	*p* = −0.04	0%	*p* = 0.82
		TAU	3	−0.31 (−0.75, −0.02)	*p* = 0.16	0%				
Exercise duration (2 sub-group analysis)	IC	<12 weeks	4	0.60 (−0.42, 1.62)	*p* = 0.25	88%	0.94 (0.35, 1.53)	*p* = 0.002	87%	*p* = 0.4
		≥12 weeks	7	1.15 (0.37, 1.93)	*p* = 0.004	88%				
	WM	<12 weeks	3	0.52 (0.14, 0.91)	*p* = 0.008	2%	0.44 (0.09, 0.78)	*p* = 0.01	55%	*p* = 0.61
		≥12 weeks	5	0.35 (−0.18, 0.88)	*p* = 0.19	70%				
	CF	<12 weeks	3	−0.53 (−0.91, −0.15)	*p* = 0.006	0%	−0.27(−0.52, −0.02)	*p* = 0.04	0%	*p* = 0.07
		≥12 weeks	3	−0.06 (−0.40, 0.27)	*p* = 0.71	0%				
Exercise intensity (2 sub-group analysis)	IC	Modetrate	5	0.76 (0.38, 1.14)	*p*<0.01	23%	0.82 (0.24, 1.40)	*p* = 0.006	86%	*p* = 0.85
		Modetrate-to-vigorous	5	0.87 (−0.22, 1.96)	*p* = 0.12	93%				
	WM	Modetrate	4	0.27 (−0.43, 0.96)	*p* = 0.45	73%	0.37 (0.01, 0.74)	*p* = 0.05	56%	*p* = 0.7
		Modetrate-to-vigorous	3	0.42 (0.06, 0.78)	*p* = 0.02	21%				
	CF	Modetrate	3	−0.33 (−0.72, 0.06)	*p* = 0.10	32%	−0.27 (−0.52, −0.02)	*p* = 0.04	0%	*p* = 0.7
		Modetrate-to-vigorous	3	−0.22 (−0.64, 0.21)	*p* = 0.32	0%				

IC, inhibitory control; WM, working memory; CF, cognitive flexibility; Open, open-skill exercise; Close, closed-skill exercise; ASD, autism spectrum disorder; ADHD, attention deficit hyperactivity disorder; NT, no treatment; TAU, treat as usual.

#### 3.4.2 Subgroup of the core domain of the executive function

As shown in Figure 3, the pooled effect size for inhibitory control was SMD = 0.94 (95% Cl: 0.35–1.53, *p* = 0.002), indicating that chronic exercise produces a substantial improvement in this domain, although heterogeneity was high (*I*^2^ = 87%). For working memory, the combined effect size was SMD = 0.44 (95% Cl: 0.09–0.78, *p* = 0.01), reflecting a moderate improvement with moderate heterogeneity (*I*^2^ = 55%). Cognitive flexibility also showed a small but statistically significant improvement (SMD = −0.27, 95% Cl: −0.52 to −0.02, *p* = 0.04), with no observed heterogeneity (*I*^2^ = 0%). Because cognitive flexibility outcomes were harmonized such that negative values indicate better performance, this finding suggests a modest beneficial effect of chronic exercise on cognitive flexibility. Overall, chronic exercise showed the largest effect on inhibitory control (SMD = 0.94), followed by a moderate effect on working memory (SMD = 0.44) and a smaller but significant beneficial effect on cognitive flexibility (SMD = −0.27, where negative values indicate improvement).

#### 3.4.3 Subgroup of intervention types

**Figure 4:**
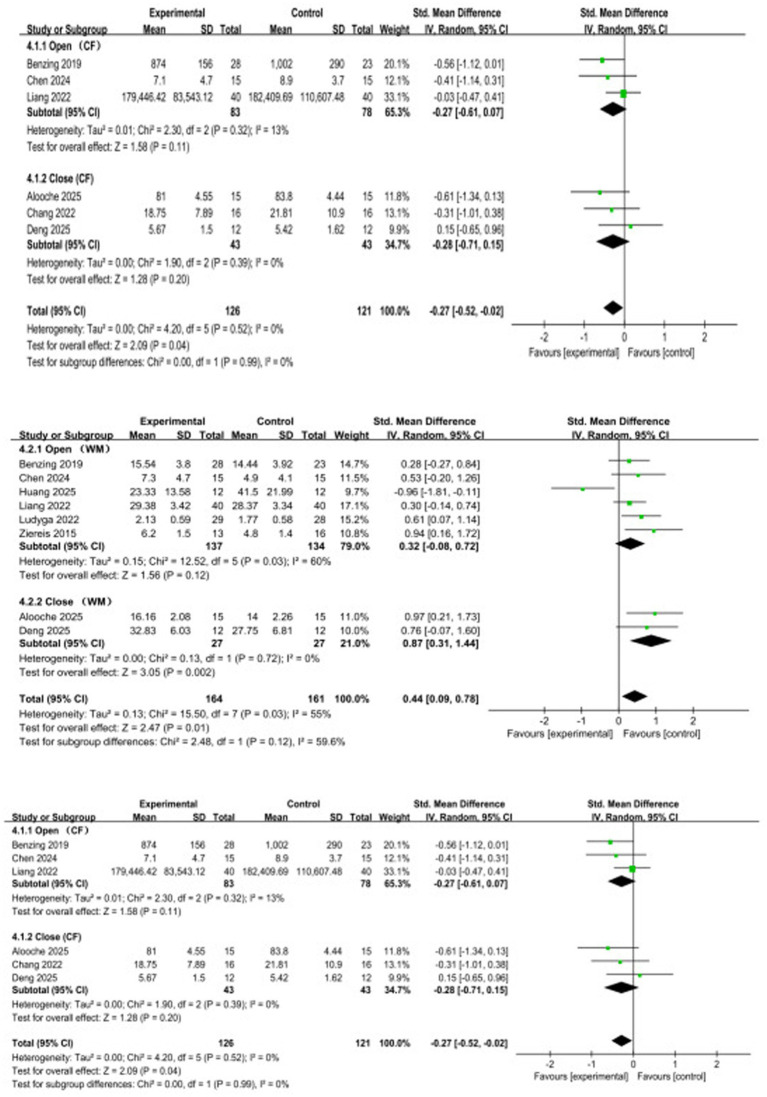
Subgroup analysis of the impact of exercise intervention types on executive functions.

To examine whether different types of motor skills exert distinct effects on various executive function subdomains, we categorized interventions into open-skill exercise—such as table tennis, taekwondo, and roller skating, which require continuous adjustment to changing environments—and closed-skill exercises, such as gymnastics and treadmill training, which involve more predictable, fixed movement patterns. Subgroup analyses were then conducted to compare the effects of these two exercise types. The specific analysis is shown in Figure 4.

Subgroup analyses suggested that intervention type may moderate effects on inhibitory control (IC). The combined effect size of open-skill exercise on inhibitory control was SMD = 0.90 (95% Cl: 0.05–1.75, *p* = 0.04), indicating a significant moderate to large effect size improvement in inhibitory control, but with high heterogeneity among studies (*I*^2^ = 91%). The combined effect size of closed-skill exercise on inhibitory control was SMD = 1.04 (95% Cl: 0.60–1.48, *p* < 0.01), indicating a significant large effect size improvement in inhibitory control, and with low heterogeneity among studies (*I*^2^ = 23%). Both types of intervention can significantly improve IC, but the effect of closed-skill exercise is more robust, with lower heterogeneity and better improvement results. The combined effect size of open-skill exercise on working memory was 0.32 (95% Cl: −0.08 to 0.72, *p* = 0.12), showing no significant improvement effect and moderate heterogeneity (*I*^2^ = 60%). Closed-skill exercise significantly improved WM (SMD = 0.87, 95% Cl: 0.31–1.44, *p* = 0.002), without heterogeneity (*I*^2^ = 0%). In terms of cognitive flexibility, open-skill exercise showed a small but significant improvement (SMD = −0.27, 95% Cl: −0.52 to −0.02, *p* = 0.04), with no heterogeneity (*I*^2^ = 0%); closed-skill exercise did not show a significant improvement effect (SMD = −0.28, 95% Cl: −0.71 to 0.15, *p* = 0.20, *I*^2^ = 0%). The results suggest that the effects of exercise type on cognitive flexibility were modest overall. Open-skill exercise was associated with a small but significant improvement, whereas closed-skill exercise did not show a statistically significant effect.

#### 3.4.4 Subgroup of intervention duration

To explore the potential moderating effect of intervention duration on the intervention outcome of chronic exercise, we divided the included studies into two subgroups: the < 12-week group and the ≥ 12-week group, and conducted subgroup analyses on IC, WM, and CF, respectively. The specific analysis is shown in Supplementary Figure S1.

In terms of IC, the combined effect size of < 12 weeks was SMD = 0.60 (95% Cl: −0.42 to 1.62, *p* = 0.25), with high heterogeneity (*I*^2^ = 88%), and did not reach statistical significance. The combined effect size of ≥12 weeks was SMD = 1.15 (95% Cl: 0.37–1.93, *p* = 0.004), with high heterogeneity (*I*^2^ = 88%), showing a significant improvement in IC. In terms of WM, the combined effect size of <12 weeks was SMD = 0.52 (95% Cl: 0.14–0.91, *p* = 0.008), with extremely low heterogeneity (*I*^2^ = 2%), demonstrating a significant improvement in WM. The combined effect size of the ≥ 12-week group (*K* = 5) was SMD = 0.35 (95% Cl: −0.18 to 0.88, *p* = 0.19), with high heterogeneity (*I*^2^ = 70%), and did not reach statistical significance. In terms of CF, the pooled effect size for interventions lasting < 12 weeks was SMD = −0.53 (95% Cl: −0.91 to −0.15, *p* = 0.006), indicating a significant improvement in cognitive flexibility, with no observed heterogeneity (*I*^2^ = 0%). By contrast, interventions lasting ≥ 12 weeks did not show a significant effect on CF (SMD = −0.06, 95% Cl: −0.40 to 0.27, *p* = 0.71). In the subgroup of exercise duration, an intervention of ≥12 weeks significantly improved IC, and an intervention of < 12 weeks significantly improved WM. In terms of CF, interventions lasting < 12 weeks showed a significant improvement, whereas interventions lasting ≥ 12 weeks did not show a statistically significant effect.

#### 3.4.5 Subgroup of intervention intensity

To examine whether exercise intensity may differentially influence the subdomains of executive function, the included studies were categorized into two intensity groups, namely moderate and moderate-to-vigorous. Subgroup analyses were then performed separately for inhibitory control, working memory, and cognitive flexibility. The detailed results are presented in Supplementary Figure S2.

In terms of IC, the combined effect size of moderate-intensity exercise was SMD = 0.76 (95% Cl: 0.38–1.14, *p* < 0.01), with low heterogeneity (*I*^2^ = 23%). The combined effect size of moderate-to-vigorous intensity exercise was SMD = 0.87 (95% Cl: −0.22 to 1.96, *p* = 0.12), but the heterogeneity was relatively high (*I*^2^ = 93%). Moderate-intensity exercise had a significant improvement effect on IC. In terms of WM, the combined effect size of moderate-intensity exercise was SMD = 0.27 (95% Cl: −0.43 to 0.96, *p* = 0.45), with high heterogeneity (*I*^2^ = 73%), and did not reach statistical significance. The combined effect size of moderate- to high-intensity exercise was SMD = 0.42 (95% Cl: 0.06–0.78, *p* = 0.02), with low heterogeneity (*I*^2^ = 21%), indicating a significant improvement in WM. Regarding CF, the combined effect size of moderate-intensity exercise was SMD = −0.33 (95% Cl: −0.72 to 0.06, *p* = 0.10), with moderate heterogeneity (*I*^2^ = 32%). The combined effect size of moderate- to high-intensity exercise was SMD = −0.22 (95% Cl: −0.64 to 0.21, *p* = 0.32), with no heterogeneity (*I*^2^ = 0%). No statistically significant improvement was found in either group. In the exercise-intensity subgroup analysis, moderate-intensity exercise was associated with a significant benefit in inhibitory control, whereas moderate-to-high-intensity exercise showed a significant effect on working memory. Neither intensity level produced a significant effect on cognitive flexibility.

#### 3.4.6 Diagnostic subtypes

To examine whether diagnostic subtype moderated the effects of chronic exercise, the included studies were classified into two subgroups—attention-deficit/hyperactivity disorder (ADHD) and autism spectrum disorder (ASD)—and separate subgroup analyses were performed for IC, WM, and CF. Detailed results are presented in Supplementary Figure S3. In terms of IC, the combined effect size of ADHD was SMD = 0.95 (95% Cl: 0.19–1.71, *p* = 0.01), with a relatively high heterogeneity (*I*^2^ = 90%). The combined effect size of ASD was SMD = 0.95 (95% Cl: 0.21–1.68, *p* = 0.01), with moderate heterogeneity (*I*^2^ = 60%). The effect sizes of the two groups were highly consistent, indicating that chronic exercise intervention significantly improved the inhibitory control function of children with ADHD and ASD to a moderate extent. In terms of WM, the combined effect size of ADHD was SMD = 0.28 (95% Cl: −0.20 to 0.75, *p* = 0.25), with high heterogeneity (*I*^2^ = 67%), and did not reach statistical significance. The combined ASD effect size was SMD = 0.75 (95% Cl: 0.31–1.19, *p* = 0.001), with no heterogeneity (*I*^2^ = 0%), demonstrating a significant improvement effect. In terms of CF, the combined effect size of ADHD was SMD = −0.25 (95% Cl: −0.57 to 0.07, *p* = 0.13), with low heterogeneity (*I*^2^ = 7%). The combined ASD effect size was SMD = −0.31 (95% Cl: −0.75 to 0.12, *p* = 0.16), with no heterogeneity (*I*^2^ = 0%). Both ADHD and ASD subgroups showed a nonsignificant trend toward improved cognitive flexibility, although neither subgroup reached statistical significance. In the subgroups of diagnostic subtypes, both children with ADHD and ASD showed significant improvement in IC, while children with ASD showed more significant improvement in WM. There was no improvement in CF in either group.

#### 3.4.7 Subgroup of control group

To explore the potential regulatory effect of the control group type on the intervention effect of chronic exercise, we divided the included studies into two subgroups: the non-intervention group (NT) and the conventional treatment control group (TAU), and conducted subgroup analyses on IC, WM, and CF, respectively. The specific analysis is shown in Supplementary Figure S4.

In terms of IC, the combined effect size of NT was SMD = 0.77 (95% Cl: 0.03–1.52, *p* = 0.04), with a relatively high heterogeneity (*I*^2^ = 89%). The combined effect size of TAU was SMD = 1.25 (95% Cl: 0.46–2.03, *p* = 0.002), with moderate heterogeneity (*I*^2^ = 74%). Both groups showed significant improvement effects. In terms of WM, the combined effect size of NT was SMD = 0.28 (95% Cl: −0.20 to 0.75, *p* = 0.25), with high heterogeneity (*I*^2^ = 67%), and did not reach statistical significance. The combined effect size of TAU was SMD = 0.75 (95% Cl: 0.30–1.19, *p* = 0.001), with no heterogeneity (*I*^2^ = 0%), showing a significant improvement effect. In terms of CF, the combined effect size of NT was SMD = −0.25 (95% Cl: −0.57 to 0.07, *p* = 0.13), with low heterogeneity (*I*^2^ = 7%), and did not reach statistical significance. The combined effect size of TAU was SMD = −0.31 (95% Cl: −0.75 to 0.02, *p* = 0.16), with no heterogeneity (*I*^2^ = 0%), and it also did not show a significant improvement effect. Both groups showed a slight trend toward improvement in CF, which was consistent with the direction of the overall CF effect (SMD = −0.27, where negative values indicate improvement).

In the control subgroup, studies with conventional treatment as the control group showed more significant improvement effects in both IC and WM, while studies using no treatment controls showed a significant effect only for inhibitory control.

### 3.5 Sensitivity analysis

Following the sequential removal of each included study and repetition of the meta-analysis, no meaningful changes were observed in either the direction or the statistical significance of the pooled effects for IC, WM, and CF, and the effect size ranges remained comparable to the original estimates (IC: 0.94, WM: 0.44, CF: −0.27). Because the included cognitive tests differed in scoring direction, outcome data were recoded before analysis so that the direction of effect was comparable across studies. After harmonization, a negative SMD for cognitive flexibility indicated better performance in the exercise group relative to the control group. This indicates that the results of this study are relatively robust and not overly influenced by individual studies. It indicates that the combined effect size in this study is stable and reliable.

### 3.6 Publication bias analysis

To assess the publication bias of the included studies, funnel plots were drawn for the three executive function subdomains of IC, WM, and CF, respectively, for intuitive judgment. The results are shown in Supplementary Figure S5. Funnel plots were visually inspected for potential publication bias. However, because the number of effect sizes was small in some domains, especially working memory and cognitive flexibility, the interpretability of these plots is limited. Therefore, publication bias cannot be ruled out.

## 4 Discussion

This meta-analysis systematically evaluated the impact of chronic exercise intervention on the executive function of children with neurodevelopmental disorders. The results indicated that chronic exercise had a moderate improvement effect on overall executive function (SMD = 0.46), especially on the enhancement of inhibitory control (SMD = 0.94). However, the effects on working memory (SMD = 0.44) and cognitive flexibility (SMD = −0.27) were smaller than those observed for inhibitory control. For cognitive flexibility, negative values indicated improvement after score harmonization, suggesting a modest but significant benefit. This finding suggests that the improvement effects of chronic exercise intervention on different subdomains of executive function vary, which may be related to the differences in the underlying neurocognitive mechanisms (13, 32). One important issue in the present meta-analysis is the substantial heterogeneity observed in the overall analysis and particularly in the inhibitory control domain. Although subgroup analyses were performed based on exercise type, intervention duration, intensity, diagnosis, and control condition, heterogeneity remained high in several subgroups. This suggests that the between-study variability was not fully explained by the factors examined in the current study. Several factors may have contributed to this heterogeneity, including differences in intervention protocols, such as exercise modality, session length, frequency, intensity, and intervention period; variation in participant characteristics, including diagnostic subtype and symptom severity; and differences in outcome assessment tools used to measure executive function. In addition, the relatively small number of included studies in some subgroup analyses may have limited the ability to detect the true sources of heterogeneity.

### 4.1 Subgroup analysis of intervention types

The classification of open-skill and closed-skill was first proposed by Poulton in 1957 (33). It was further refined by scholars such as Gentile. Open-skill require athletes to continuously monitor environmental changes, make quick decisions, and flexibly adjust movement strategies, which poses high demands on attention shift, inhibitory control, and cognitive flexibility (34, 35). In contrast, closed-skill exercise refer to motor tasks performed in a relatively stable and predictable environment, with relatively fixed action patterns. Participants can pre-plan and repeatedly execute the same sequence of actions (34, 36). In the present study, closed-skill exercise was associated with significant effects on inhibitory control and working memory, whereas open-skill exercise was associated with significant effects on inhibitory control and a small but significant effect on cognitive flexibility. This finding is highly consistent with the results of recent meta-analyses of the ADHD population (4, 37). Qiu et al. conducted a systematic review and meta-analysis involving 578 individuals with ADHD and reported that closed-skill exercise was associated with significant improvements across several executive function domains, including inhibitory control (SMD = −1.00), cognitive flexibility (SMD = −1.33), and working memory (SMD = −0.85). They also found that open-skill exercise produced beneficial effects on inhibitory control (SMD = −1.98) and cognitive flexibility (SMD = −0.97) (4). Closed-skill exercise appeared to show more consistent benefits for inhibitory control and working memory, possibly because of its structured, repetitive, and less distracting task demands. In contrast, the beneficial effect of open-skill exercise on cognitive flexibility may be related to the cognitively demanding nature of these activities, which require continuous environmental monitoring, rapid decision-making, and flexible updating of motor responses (34). These task characteristics may be particularly relevant to cognitive flexibility, although this interpretation remains tentative. The systematic review by Liu et al. also confirmed that there is a significant interaction between exercise types and executive function subdomains, suggesting that exercise types should be carefully designed for different executive function subdomains. Additional research confirms that the type of motor skill can influence executive function, showing that both open-skill and sequential skills positively affect multiple executive function domains in children with atypical development (13). Moreover, recent analyses indicate that activities combining cognitive engagement, such as multi-component ball games, produce greater improvements in cognitive function among children with neurodevelopmental disorders (32).

### 4.2 Subgroup analysis of intervention duration

The influence of intervention duration on each subdomain of executive function shows a differentiated pattern: Intervention < 12 weeks significantly improves working memory (SMD = 0.52), while motor intervention ≥ 12 weeks has a better inhibitory control effect (SMD = 1.15); Combined with this discovery in terms of cognitive flexibility, it suggests that there are optimal intervention time windows in different executive function subdomains. A systematic review indicated that both the number of intervention weeks and the total intervention duration are important moderators of executive function, with effects differing across subdomains (13). Randomized controlled trials further demonstrate that a 12-week intervention positively influences inhibitory control and planning abilities (38). At the same time, some studies suggest that interventions shorter than 10 weeks can also effectively enhance executive function in children with atypical development, implying a possible nonlinear relationship between intervention duration and effect size (39). The significant improvement in working memory caused by short-term intervention may be related to the immediate effects of acute exercise. Walters et al. found that a single exercise can improve working memory, while inhibitory control requires a longer period of intervention and accumulation to form stable neural adaptation. From a developmental perspective, Best pointed out that working memory develops rapidly in the preschool period, while inhibitory control continues to develop into adolescence. This difference in developmental trajectories may lead to different sensitivities of different subdomains to the duration of intervention (40). The finding that interventions lasting < 12 weeks were associated with improved cognitive flexibility may suggest that this domain is responsive to exercise under certain conditions, although the effect size was modest and based on a limited number of studies. Because cognitive flexibility develops relatively late and is measured with diverse tools, this result should be interpreted cautiously and requires further confirmation. The subgroup findings tentatively suggest that shorter interventions may be sufficient for working memory, whereas longer interventions may be more relevant for inhibitory control; however, these patterns require confirmation in adequately powered head-to-head trials. Future research should adopt a multi-time-point measurement design and further conduct experimental analysis and exploration of short-term and long-term interventions through larger sample experiments. It should be emphasized that interventions lasting 8–12 weeks do not necessarily represent chronic exercise in the strictest sense. In this review, the term was used to distinguish repeated exercise programs from acute exercise. Therefore, findings from shorter interventions should be interpreted as evidence for structured repeated exercise rather than definitive evidence for long-term or six-month chronic exercise effects. Future trials should include intervention periods of 6 months or longer to determine whether exercise produces sustained improvements in executive function.

### 4.3 Subgroup analysis of intervention intensity

The subgroup analysis by exercise intensity showed that moderate-intensity exercise was associated with a significant improvement in inhibitory control (SMD = 0.76), while moderate-to-high-intensity exercise appeared to be more beneficial for working memory (SMD = 0.42). These findings suggest that different executive function subdomains may vary in their sensitivity to exercise intensity. This observation aligns with several recent studies and offers mechanistic insights from multiple perspectives. A meta-analysis of fMRI studies using activation likelihood estimation highlighted the brain-region specificity of exercise effects on executive function: chronic exercise increased activation in the left precuneus and right subfrontal gyrus during inhibitory control tasks, while working memory tasks showed significant changes in the right thalamus and right paracentral lobule (41). These differential neural activation patterns may partly explain these patterns for the varying sensitivity of executive function subdomains, suggesting that core regions associated with inhibitory control respond optimally to moderate-intensity exercise, whereas regions involved in working memory require higher-intensity stimulation for full activation. A network meta-analysis compared the effects of different types of exercise on the cognitive function of children with NDDs and found that physical and mental exercise had the most significant improvement in attention (SMD = 1.91), exercise games had a prominent effect on memory (SMD = 0.97) and executive function (SMD = 0.94), and group exercise had a stable improvement in all cognitive domains. It is worth noting that simple aerobic exercise did not show significant effects, suggesting that the exercise components involved in cognition may be more crucial than the exercise intensity itself (42). The study also pointed out that the intensity and type of exercise intervention should be designed to match the target cognitive function. A meta-analysis of inhibitory control in children with ASD found that regimens with a single duration of no more than 45 min, a frequency of more than 2 times per week, and an intervention period of no more than 4 weeks tended to produce better improvement effects. This suggests that, apart from intensity, there are complex interactions among frequency, single duration, and period (43). Future research still needs to conduct a comprehensive analysis of the intervention from multiple perspectives and its complex cross-effects. This pattern is roughly consistent with the previous meta-analysis evidence of ADHD, that is, the impact of physical activity on executive function may be potentially modulated by factors such as intervention intensity, type of motor skills, conversation frequency and exercise volume (44).

### 4.4 Analysis of diagnostic subtypes

Before interpreting the diagnostic-subtype findings, it should be noted that ADHD and ASD differ in their core clinical manifestations, developmental characteristics, and executive-function profiles. Therefore, the two diagnostic groups should not be regarded as fully equivalent. In the present meta-analysis, they were analyzed within the broader framework of neurodevelopmental disorders because both conditions are commonly associated with executive-function impairments and both met the predefined eligibility criteria. However, the evidence base was unbalanced, with more studies involving children with ADHD than children with ASD. Thus, the diagnostic-subtype results should be interpreted cautiously, and the ASD-related findings should be considered exploratory rather than confirmatory.

The diagnostic-subtype analysis suggested that exercise intervention may improve inhibitory control in both ADHD and ASD samples (ADHD: SMD = 0.95; ASD: SMD = 0.95). However, because the ASD subgroup contained only a small number of studies, this result should be interpreted as preliminary. The finding that children with ASD showed a relatively larger improvement in working memory (SMD = 0.75) may indicate a potential diagnostic difference in response to exercise intervention, but this interpretation remains exploratory and requires confirmation in future trials with larger ASD samples. From the perspective of neural mechanisms, the effects of exercise intervention were revealed. Evidence from electroencephalography, functional magnetic resonance imaging, diffusion tensor imaging, and functional near-infrared spectroscopy suggests that exercise interventions may alter neural activity, as reflected in more normalized cortical arousal in individuals with ADHD and strengthened social brain connectivity in those with ASD. These changes may also be associated with more efficient executive function processing in individuals with neurodevelopmental disorders (45). More research has found that physical activities and exercise intervention programs for children with neurodevelopmental disorders not only enhance executive function but also simultaneously improve social skills and quality of life (32). The core deficiency of ADHD lies in inhibitory control and attention maintenance, while ASD is more characterized by deficits in social, emotional, and cognitive flexibility. One possible explanation is that children with ASD may respond differently to exercise programs that combine motor coordination, structured routines, and cognitive engagement. However, this explanation remains speculative because of the small number of ASD studies included (46). Overall, these findings suggest that diagnostic subtype may moderate the effects of exercise intervention on executive function. Nevertheless, because the ADHD and ASD subgroups were not equally represented, the pooled results should not be interpreted as evidence that exercise has identical effects across different neurodevelopmental disorders.

### 4.5 Analysis of the control group

The subgroup analysis by control condition indicated that the effects of chronic exercise intervention on executive function may differ according to the type of comparison group. In studies using no-treatment controls, the beneficial effect of exercise appeared to be more evident, which may be related to the clearer contrast between participation in structured exercise and the absence of additional intervention. In studies using treatment-as-usual controls, the observed effect may suggest that exercise intervention provides additional benefits when combined with usual care. However, this finding should be interpreted cautiously, as usual care was not identical across studies and may have varied in content, frequency, and intensity. Such differences in control conditions may partly explain the heterogeneity across the included studies (47).

## 5 Limitations

This study has several limitations. First, only 13 randomized controlled trials involving 527 children were included, and the evidence base was dominated by ADHD samples, with only three studies involving ASD. Therefore, the overall findings should be interpreted primarily in relation to ADHD, whereas the ASD-related findings remain exploratory. Second, although the term “chronic exercise intervention” was used to distinguish repeated exercise programs from acute exercise, several included studies lasted only 8–12 weeks. These shorter interventions should not be interpreted as evidence for strictly long-term or six-month chronic exercise effects. Third, substantial heterogeneity was observed in the overall analysis and in several subgroup analyses, particularly for inhibitory control, which may reduce the stability of the pooled estimates.

## 6 Implication for future research

This study offers the following implications for future research: First, the exercise intervention program needs to be further optimized. This study found that closed-skill exercises appeared to be associated with more consistent benefits for inhibitory control and working memory, whereas open-skill exercise may also be beneficial for cognitive flexibility. Future trials should directly compare exercise types to clarify domain-specific effects. In the future, randomized controlled trials should be conducted directly comparing different types, intensities, and durations of exercise to determine the optimal exercise plans for different subdomains of executive function. Second, the implementation methods of intervention should be expanded. Exploring remote exercise intervention models based on network or digital medical platforms to break through the limitations of time and space and expand the beneficiary population is of great practical significance, especially at present. Thirdly, it is necessary to strengthen the comparative research between exercise intervention and other treatment methods. At present, most studies use no intervention or conventional treatment as controls. In the future, head-to-head comparisons should be designed with non-pharmaceutical treatments such as drugs, cognitive training, and neurofeedback to clarify the unique value and clinical positioning of exercise intervention. Finally, it is necessary to refine the analysis of diagnostic subtypes. Research has found that there are differences in the responses of children with ADHD and ASD in various subdomains of executive function. Intervention studies targeting the ASD population and children aged 3 to 6 are extremely limited. Given the early neural plasticity, more high-quality studies on ASD populations should be included in the future to further verify the conclusions and innovate. In addition, it is recommended to extend the follow-up period to assess the long-term maintenance of the intervention effect and provide a more sufficient evidence-based basis for clinical practice.

## 7 Conclusion

The results suggest that chronic exercise intervention, operationally defined in this review as repeated structured exercise programs rather than acute single-session exercise, may improve executive function in children with neurodevelopmental disorders, particularly inhibitory control. Smaller beneficial effects were also observed for working memory and cognitive flexibility; however, the evidence for cognitive flexibility remains limited and should be interpreted with caution. Although exercise appears to benefit inhibitory control, its effects should not be overinterpreted, and further high-quality studies are needed before firm clinical recommendations can be made (37). Moreover, because the included evidence was dominated by ADHD studies and only a small number of trials involved ASD, the pooled findings should be generalized cautiously and should not be interpreted as representing the full spectrum of neurodevelopmental disorders. Further subgroup analyses suggested that intervention type, duration, exercise intensity, diagnostic subtype, and control condition may influence the intervention effects. Among these factors, closed-skill exercise appeared to show more stable benefits for inhibitory control and working memory. Relatively longer interventions were associated with greater improvements in inhibitory control, although interventions lasting 8–12 weeks should not be interpreted as evidence for strictly long-term chronic exercise effects. Moderate-intensity exercise appeared to be associated with improvements in inhibitory control, whereas moderate- to high-intensity exercise showed a potential benefit for working memory; however, these intensity-related findings should be interpreted cautiously because of the limited number of studies in each subgroup. Overall, chronic exercise intervention may serve as a promising supplementary non-pharmacological approach for improving executive function, but its effects may vary across executive-function subdomains and diagnostic groups. Future larger-sample, higher-quality, and more standardized randomized controlled trials are needed, particularly studies with intervention periods of 6 months or longer, to clarify the optimal exercise prescription and determine whether the observed benefits can be sustained over time.

## Data Availability

The original contributions presented in the study are included in the article/Supplementary material, further inquiries can be directed to the corresponding author.
