# Supplementary figures and images for: The influence of chronic exercise intervention on the executive function of children with neurodevelopmental disorders: a meta-analysis
Source: Front Neurol. 2026 Jun 29;17:1827768. doi: 10.3389/fneur.2026.1827768 (PMC13357176; doi:10.3389/fneur.2026.1827768)

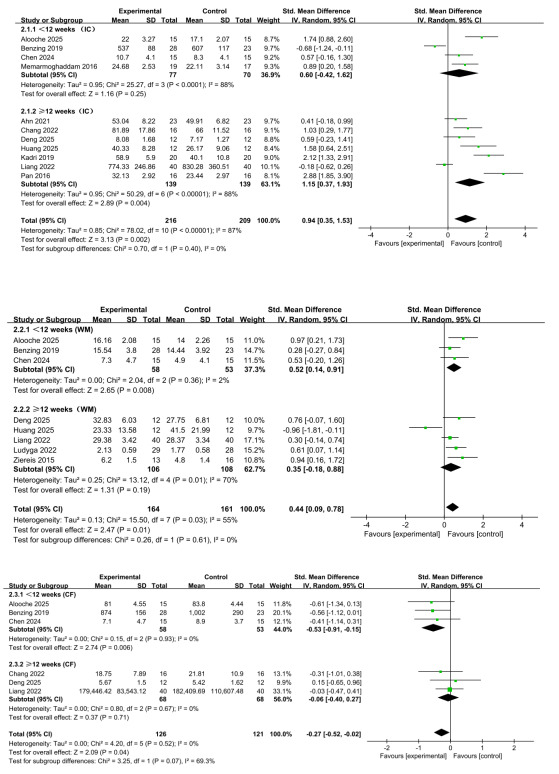

Supplement: Supplementary file 1 [file Image_1.JPEG]

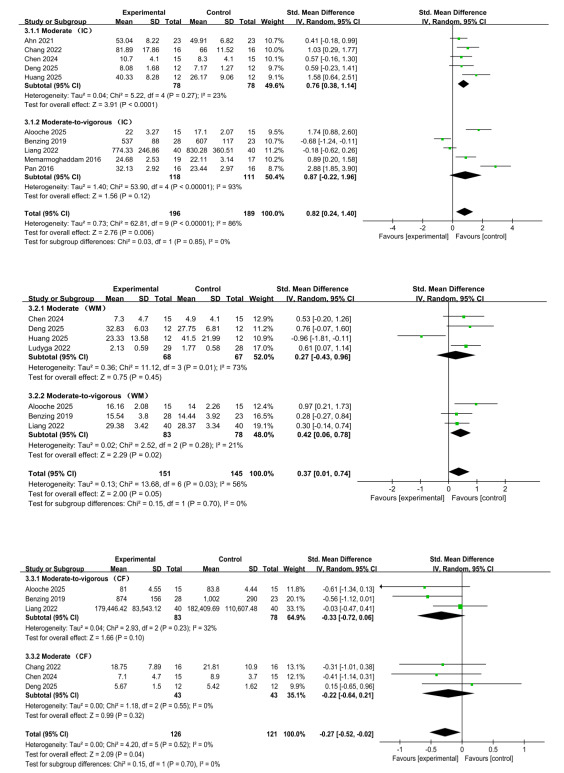

Supplement: Supplementary file 2 [file Image_2.JPEG]

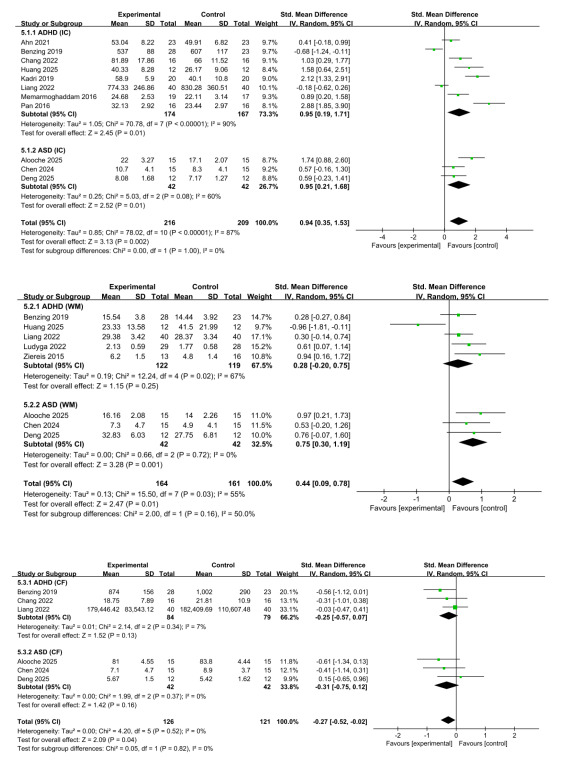

Supplement: Supplementary file 3 [file Image_3.JPEG]

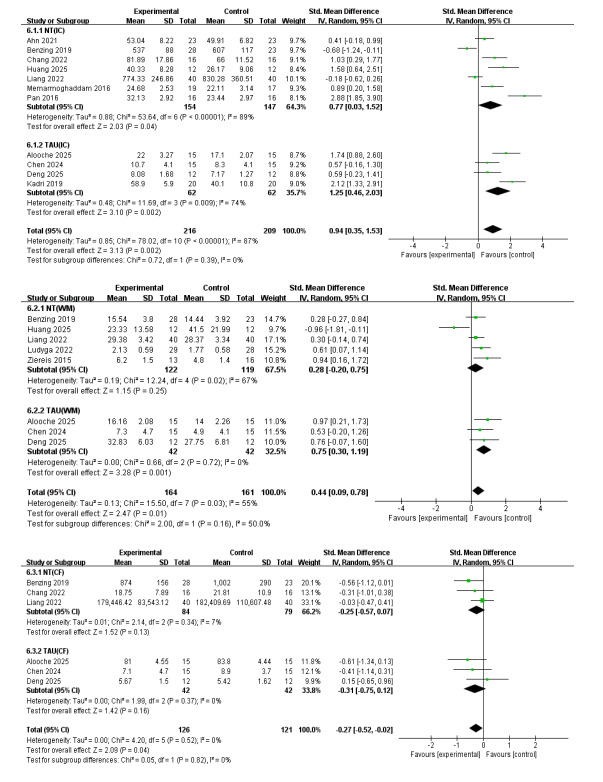

Supplement: Supplementary file 4 [file Image_4.JPEG]

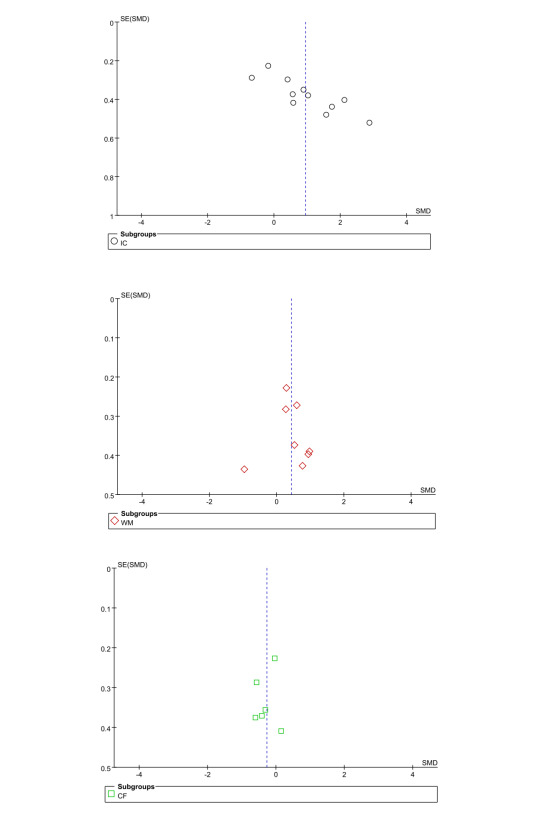

Supplement: Supplementary file 5 [file Image_5.JPEG]
